# Sarcomatoid variant urothelial carcinoma of the bladder: a systematic review and meta-analysis of the clinicopathological features and survival outcomes

**DOI:** 10.1186/s12935-020-01626-9

**Published:** 2020-11-14

**Authors:** Liangyou Gu, Qing Ai, Qiang Cheng, Xin Ma, Baojun Wang, Qingbo Huang, Xintao Li, Peng Zhang, Kan Liu, Xupeng Zhao, Hongzhao Li, Xu Zhang

**Affiliations:** 1 Department of Urology, the Third Medical Centre, Chinese PLA General Hospital, Beijing, China; 2 Department of Urology, Air force specialty medical center, Beijing, China; 3 School of Medicine, Nankai University, Tianjin, China

**Keywords:** Bladder cancer, Sarcomatoid, Survival, Urothelial carcinoma, Variant histology

## Abstract

**Background:**

A systematic review and meta-analysis was performed to compare the clinicopathological features and survival outcomes between sarcomatoid variant (SV)-urothelial carcinoma of the bladder (UCB) and conventional UCB (C-UCB).

**Methods:**

A comprehensive search of PubMed, Embase, and Cochrane Library was performed. Endpoints included clinicopathological features and survival outcomes (overall survival [OS], cancer-specific survival [CSS], and progression-free survival [PFS]). The survival benefits of neoadjuvant chemotherapy (NAC) or adjuvant chemotherapy (AC) for SV-UCB also have been studied.

**Results:**

A total of 8 observational studies were included. Patients with SV-UCB had a higher rate of ≥ stage pT3 (odds ratio [OR], 2.06; 95% confidence interval [CI], 1.64–2.59; p < 0.001) and a lower rate of concomitant carcinoma in situ (OR, 0.25; 95% CI, 0.09–0.72; p = 0.010). The other clinicopathological variables were similar between SV-UCB and C-UCB. With unadjusted data, patients with SV-UCB had a significant inferior OS (HR, 1.24; 95% CI, 1.07–1.44; p = 0.004) and CSS (HR, 2.08; 95% CI, 1.63–2.66; p < 0.001). However, after adjusted, SV-UCB had worse OS (HR, 1.41; 95% CI, 0.95–2.08; p = 0.090) and CSS (HR, 1.54; 95% CI, 0.95–2.52; p = 0.080) approaching the borderline of significance. For SV-UCB, NAC (HR, 0.73; 95% CI, 0.51–1.05; p = 0.090) and AC (HR, 0.88; 95% CI, 0.66–1.17; p = 0.370) seemed to have no benefit on OS.

**Conclusions:**

Compared to C-UCB, SV-UCB was associated with more advanced disease and more inferior OS and CSS. NAC and AC had no survival benefit for SV-UCB.

## Introduction

Urothelial carcinoma (UC) is the most common histologic type of bladder cancer. Around 75% of bladder cancers are classified as pure UC, and the remaining 25% are urothelial and nonurothelial histological variants [1]. Histological variants refer to different proportions of tumors occurring in the urinary tract, part of the same tumor as pure UC or in its pure form, which identified on pathological sections. The 2016 WHO classification of tumors of the urinary tract detailly described the variant morphologies [2].

Sarcomatoid variant (SV) is a rare histologic variant of UC and is estimated to account for 0.1%–0.3% of all urothelial carcinoma of the bladder (UCB) [3]. Sarcomatoid variant urothelial carcinoma of the bladder (SV-UCB) is characterized by the presence of components of two-phase malignancy, there is morphological and/or immunohistochemical evidence of epithelial and mesenchymal differentiation. [4]. For most cases with SV-UCB, the epithelial component is UC. However, squamous cell and small cell carcinoma components have also frequently been reported [5]. Malignant spindle cell components are usually undifferentiated high-grade sarcomas. Allogenic components are present in the form of rhabdomyosarcoma, chondrosarcoma, liposarcoma, and osteosarcoma [4]. Although SV-UCB was first reported as early as 1972, this disease was mainly described by single-center studies in case reports or series. More recently, a few studies have analyzed the survival outcomes of UCB with variant histology (including SV-UCB) using large disease databases [6, 7].

Many evidences supported that SV-UCB tended to present at an advanced stage and was associated with poor long-term survival [3, 8, 9]. However, single-center study failed to identify a worse prognosis when compared with conventional UCB (C-UCB) [10]. Moreover, compared with C-UCB, Moschini et al. [11] and Monn et al. [12] have found that sarcomatoid variant was not an independent predictor of poor prognosis. The current body of data of SV-UCB is limited to case reports or series, and inconsistent results preclude full understanding of this disease.

Presently, the marked increase in the incidence of histological variation mainly attributes to pathologists’ awareness, increased recognition and improved reporting [13, 14]. The histology of variation has important diagnostic, prognostic and therapeutic significance. Accurate diagnosis allows risk stratification, prognosis determination, and guiding treatment decisions. Nevertheless, due to the limited data and inconsistent results, the behavior of SV-UCB and its treatment guidelines are not well characterized. Additionally, due to its rarity, it is unlikely to address this issue with large clinical trials. Hence, we aimed to systematically review the relevant literatures and perform meta-analyses with available data comparing the clinicopathological features and survival outcomes between SV-UCB and C-UCB.

## Evidence acquisition

The study was performed according to the Preferred Reporting Items for Systematic Reviews and Meta-analysis (PRISMA) criteria (Additional file 1), and the protocol was registered (CRD42020182608).

### Search strategy

A systematic literature searching was performed in the Pubmed, Embase, and Cochrane Library on April, 2020 to identify potential studies. The used terms were as following: (“bladder cancer” OR “bladder tumor” OR “bladder carcinoma” OR “bladder urothelial carcinoma”), (“sarcomatoid” OR “sarcomatoid variant” OR “sarcomatoid carcinoma” OR “carcinosarcoma”), and relevant variants. The language of literatures was restricted to English. Two authors independently screened the titles and abstracts of potential literatures and assessed the full-text articles. In case of the disagreement on inclusion, it was checked and decided by a senior researcher.

### Inclusion criteria and study eligibility

The present study included literatures embracing comparative data about clinicopathological features and survival outcomes between patients with SV-UCB and C-UCB. The studies embracing data about the survival benefit of neoadjuvant chemotherapy (NAC) or adjuvant chemotherapy (AC) for patients with SV-UCB were also included. There were no restrictions on study design, all types of observational studies were selected. Exclusion criteria included the following items: (1) cell or animal research; (2) studies out of scope (comparisons of the clinicopathological features and survival outcomes between SV-UCB and C-UCB); (3) studies didn’t provide extractable data; (4) non-original articles, such as reviews, letters, editorials, comments; (5) gray literatures, such as conference abstracts.

### Data extraction

The primary outcomes were differences in clinicopathological features (pathological T3 and higher, high grade, concomitant carcinoma in situ, positive lymph node, positive surgical margin) and survival outcomes (overall survival [OS], cancer-specific survival [CSS], progression-free survival [PFS]). The secondary outcomes were differences in rate of NAC or AC administration, and the survival benefit (overall survival) of NAC or AC for patients with SV-UCB.

Two authors independently reviewed the included literatures and extracted required data. In case of the disagreement, it was checked and decided by a senior researcher. A pre-designed table was used, including study features (name of first author, year of publication, patients’ region and period, study design, sample size, treatment), clinical characteristics (patient age, gender, rate of NAC and AC), pathological features, and survival outcomes. The hazard ratios (HRs) and 95% confidence intervals (CIs) for all survival outcomes were extracted when provided, or calculated with the data from literatures using the method reported by Tierney et al. [15].

### Study quality assessments and quality of evidence

The Newcastle–Ottawa Scale was used to assess study quality [16]. The certainty of evidence was rated using The Grading of Recommendations, Assessments, Developments, and Evaluation (GRADE) system [17], which included the following five criteria, study design, risk of bias, inconsistency and precision of results, and indirectness. The certainty of the evidence of each meta-analysis was attributed to four levels.

### Statistical analysis

The differences in clinical and pathological characteristics were assessed with the odd ratios (ORs) and 95% CIs. The differences in survival outcomes were assessed with the HRs and 95% CIs. For each meta-analysis, the Cochrane Q statistic and *I*^*2*^ statistic were used to assess the statistical heterogeneity among included literatures. A p value lower than 0.05 in Cochrane Q statistic or value of *I*^*2*^ higher than 50% was deemed as significant heterogeneity, a random-effect model was used at this time. Otherwise, a fixed-effect model was used. Funnel plot was used to assess publication bias. All analyses were performed with Review Manager v.5.3 (The Cochrane Collaboration, Denmark). A two-sides p value lower than 0.05 was deemed as statistically significant.

## Evidence synthesis

### Data retrieval process

**Fig. 1:**
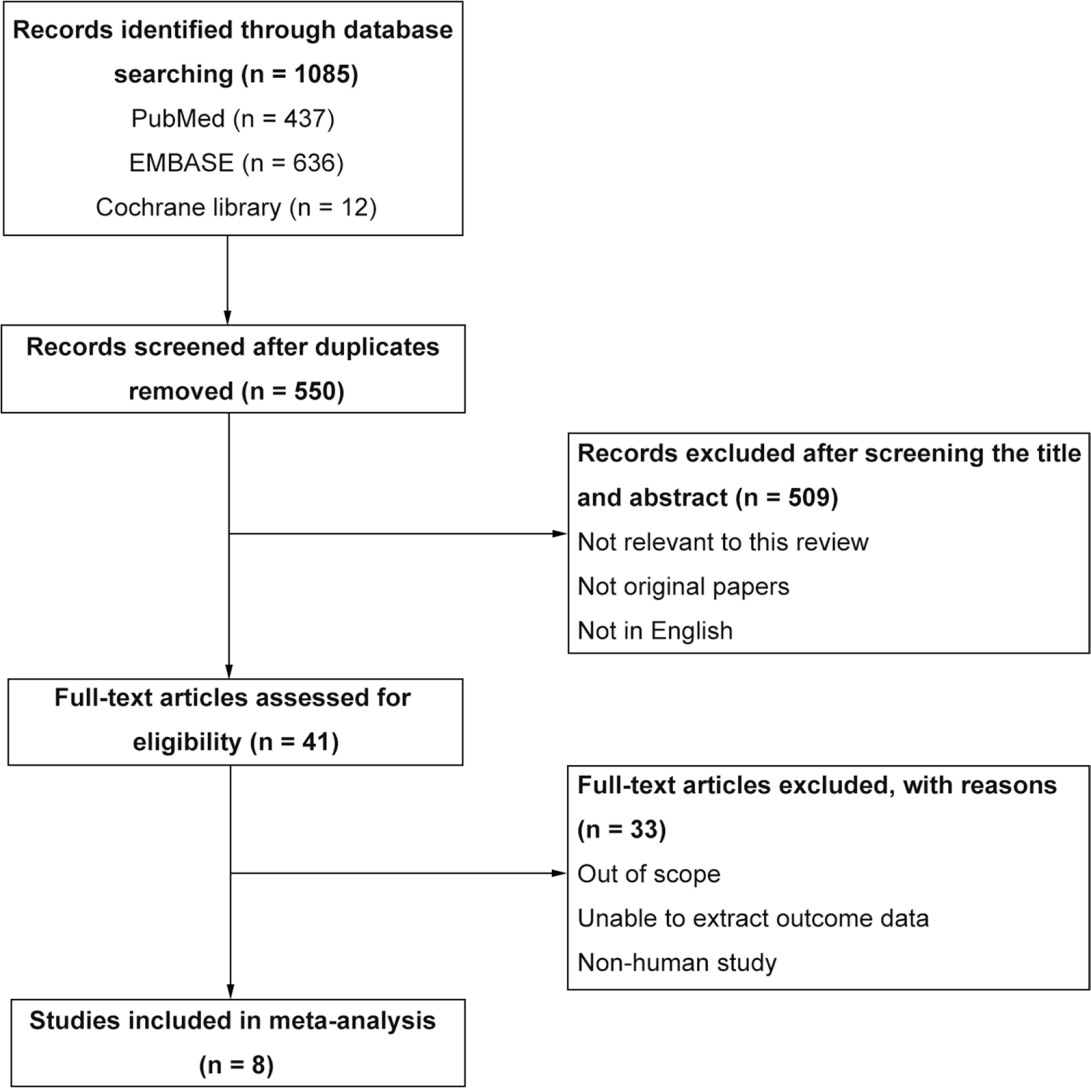
Flowchart of literature searching and inclusion

The process of literature searching and study inclusion was present in Fig. 1. The primary searching in three databases retrieved 1085 records. After excluding duplicates, 550 literatures remained. Based on inclusion and exclusion criteria, 509 records were excluded by screening the title and abstract. Forty-one full-text articles were assessed for eligibility, 33 of them were excluded due to out of scope, unable to extract outcome data, non-human study. Lastly, 8 literatures were included in the present study [3, 6, 7, 9–12, 18]. All studies were retrospective observational studies, the detailed characteristics were shown in Table 1. The clinical and pathological characteristics of included patients were present in Table 2.

**Table 1:** Characteristics of studies included in the current study

Study (first author, year)	Country	Study design, Year of inclusion	Treatment	Definition of SV-UCB	Number of patients	Reported outcomes for comparison between-SV-UCB and C-UCB	Data on NAC/AC benefit for SV-UVB	Data used for the following meta-analysis
Berg (2019)	United States and Puerto Rico	Retrospective, 2004–2015	Radical cystectomy	Any component of sarcomatoid variant	SV-UCB: 388 C-UCB: 13,210	AC, unadjusted OS	AC benefit on OS	AC, unadjusted OS, AC benefit on OS
Robinson (2018)	United Kingdom	Retrospective, 1999–2015	Radical cystectomy	Any component of sarcomatoid variant	SV-UCB: 12 C-UCB: 230	pT stage, high grade, concomitant CIS, pN + , surgical margin + , unadjusted OS/CSS/PFS	NR	pT stage, high grade, concomitant CIS, pN + , surgical margin + , unadjusted OS/CSS/PFS
Vetterlein (2017)	United States and Puerto Rico	Retrospective, 1998–2012	Radical cystectomy	Any component of sarcomatoid variant	SV-UCB: 304	NR	NAC benefit on OS	NAC benefit on OS
Sui (2017)	United States	Retrospective, 2004–2013	Radical cystectomy	Any component of sarcomatoid variant	SV-UCB: 220	NR	NAC and AC benefit on OS	NAC and AC benefit on OS
Moschini (2017)	Italy	Retrospective, 1990–2013	Radical cystectomy	Sarcomatoid variant histology without any other variant histology	SV-UCB: 21 C-UCB: 729	NAC, AC, pT stage, concomitant CIS, pN + , surgical margin + , adjusted OS/ CSS/PFS	NR	NAC, AC, pT stage, concomitant CIS, pN + , surgical margin + , adjusted OS/CSS/PFS
Monn (2015)	United States	Retrospective, 2008–2013	Radical cystectomy	Any component of sarcomatoid variant, regardless of overall percentage. In mixed variant histology, sarcomatoid variant with the highest percentage present	SV-UCB: 15 C-UCB: 462	NAC, AC, pT stage, pN + , surgical margin + , adjusted OS	NR	NAC, AC, pT stage, pN + , surgical margin + , adjusted OS
Wang (2011)	United States	Retrospective, 1997–2011	Radical cystectomy, TURBT, etc	Any component of sarcomatoid variant	SV-UCB: 14 C-UCB: 319	high grade	NR	high grade
Wright (2007)	United States	Retrospective, 1988–2003	Radical cystectomy, TURBT, etc	Any component of sarcomatoid variant	SV-UCB: 301 C-UCB: 46,515	pT stage, unadjusted CSS, adjusted OS/CSS	NR	pT stage, unadjusted CSS, adjusted OS/CSS

*SV-UVB* sarcomatoid variant urothelial carcinoma of the bladder; *C-UCB* conventional urothelial carcinoma of the bladder, *NAC* neoadjuvant chemotherapy, *AC* adjuvant chemotherapy, *OS* overall survival, *CIS* carcinoma in situ, *CSS* cancer-specific survival, *PFS* progression-free survival, *NR* not reported, *TURBT* transurethral resection of bladder tumor

**Table 2:** Comparison of the clinicopathological features between PV-UCB and C-UCB

Study (first author, year)	Groups	n	Age at surgery, year	Clinical characteristics			Pathologic characteristics						Survival outcomes (Overall survival)
				Male, n (%)	NAC, n (%)	AC, n (%)	≤ pT2, n (%)	≥ pT3, n (%)	High grade, n (%)	cCIS, n (%)	pN + , n (%)	Surgical margin + , n (%)	
Berg (2019)	SV-UCB	388	NR	NR	NR	79 (20.4)	NR	NR	NR	NR	NR	NR	Unadjusted HR (RC), 1.20; 95% CI 1.01–1.43 Unadjusted HR (RC + AC), 1.50; 95% CI 1.08–2.08
	C-UCB	13,210	NR	NR	NR	3053 (23.1)	NR	NR	NR	NR	NR	NR	
Robinson (2018)	SV-UCB	12	Mean 65.3	9 (75.0)	NR	NR	7 (58.3)	5 (41.7)	10 (83.3)	2 (16.7)	1 (8.3)	0 (0.0)	Unadjusted HR, 0.88; 95% CI 0.40–1.97
	C-UCB	230	Mean 67.7	185 (80.4)	NR	NR	140 (60.9)	90 (39.1)	213 (92.6)	105 (45.7)	51 (22.2)	21 (9.1)	
Vetterlein (2017)	SV-UCB	304	Mean 67.7	185 (80.4)	47 (15.5)	NR	NR	NR	NR	NR	NR	NR	NR
Sui (2017)	SV-UCB	220	Mean 70.4 (12.8)	NR	49 (22.3)	31 (14.1)	NR	NR	NR	NR	NR	23 (10.3)	NR
Moschini (2017)	SV-UCB	21	Median 72 (60–78)	16 (76.2)	0 (0.0)	2 (9.5)	7 (33.3)	14 (66.7)	NR	2 (9.5)	7 (33.3)	1 (4.8)	Adjusted HR, 1.14; 95% CI 0.55–2.23
	C-UCB	729	Median 68 (61–75)	613 (84.1)	18 (2.5)	107 (14.7)	308 (42.2)	421 (57.7)	NR	211 (28.9)	264 (36.2)	60 (8.2)	
Monn (2015)	SV-UCB	15	Mean 62.9 (13)	9 (60.0)	0 (0.0)	4 (26.7)	5 (33.3)	10 (66.7)	NR	NR	3 (20.0)	1 (6.7)	Adjusted HR, 1.07; 95% CI 0.43–2.69
	C-UCB	462	Mean 66.6 (11)	364 (78.8)	65 (14.1)	45 (9.7)	330 (71.4)	132 (28.6)	NR	NR	85 (18.4)	45 (9.7)	
Wang (2011)	SV-UCB	14	Median 63 (45–93) ^R^	12 (85.7)	NR	NR	NR	NR	14 (100.0)	NR	NR	NR	NR
	C-UCB	319	Median 71 (32–92) ^R^	243 (76.2)	NR	NR	NR	NR	182 (57.1)	NR	NR	NR	
Wright nn	SV-UCB	301	NR	193 (64.1)	NR	NR	137 (45.5)	93 (30.9)	NR	NR	NR	NR	Adjusted HR (SaC), 1.18; 95% CI 0.91–1.52 Adjusted HR (CS), 2.00; 95% CI 1.65–2.41
	C-UCB	46,515	NR	34,816 (75.0)	NR	NR	33,721 (72.5)	8219 (17.7)	NR	NR	NR	NR	

*SV-UVB* sarcomatoid variant urothelial carcinoma of the bladder, *C-UCB* conventional urothelial carcinoma of the bladder, *NAC* neoadjuvant chemotherapy, *AC* adjuvant chemotherapy, *cCIS* concomitant carcinoma in situ, *NR* not reported, *HR* hazard ratio, *RC* radical cystectomy, *CI* confidence interval, *Sac* sarcomatoid carcinoma, *CS* carcinosarcoma

### Clinicopathological outcomes

**Fig. 2:**
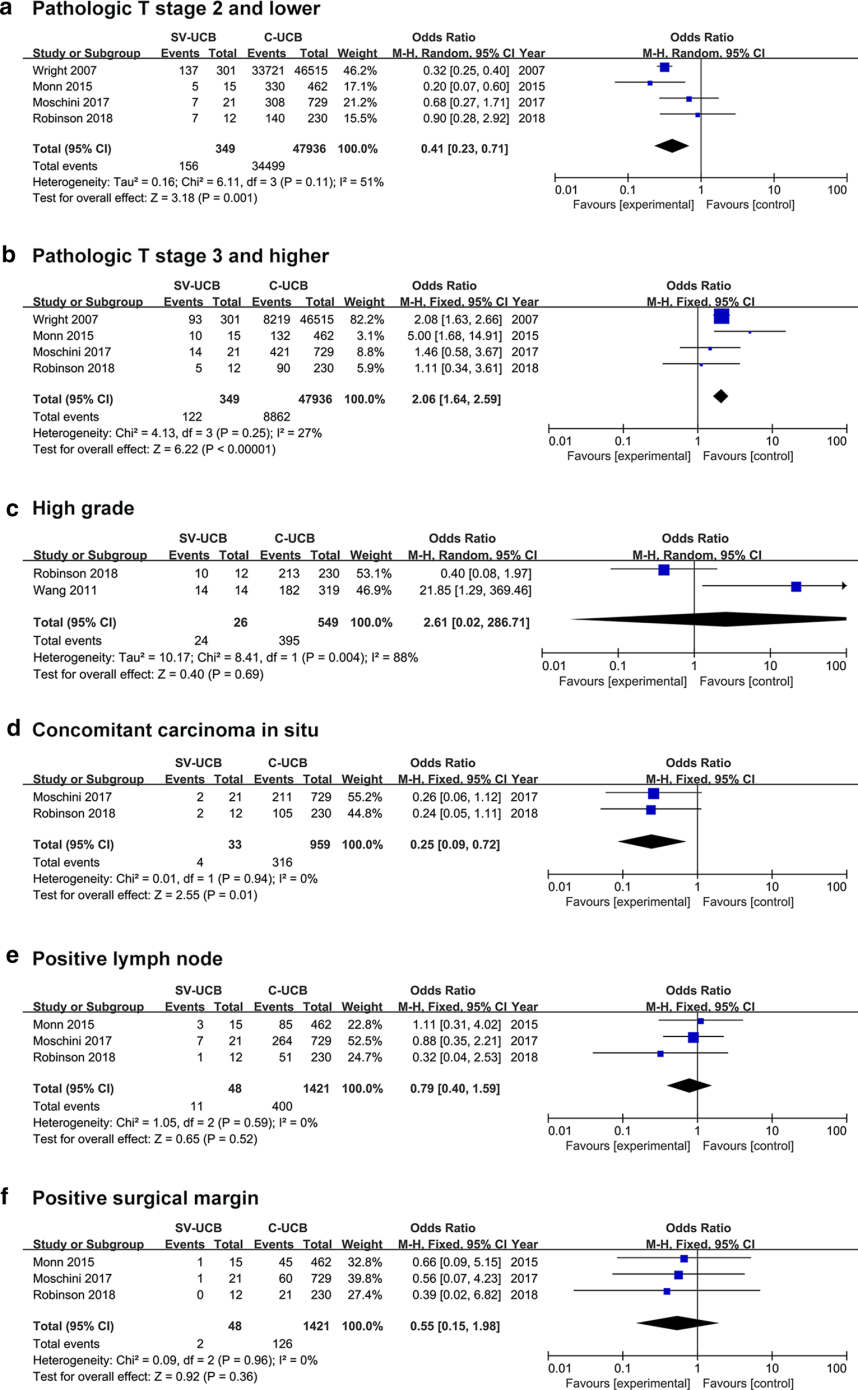
Forest plots of the clinicopathological outcomes. **a** pathological T stage 2 and lower, **b** pathological T stage 3 and higher, **c** high grade, **d** concomitant carcinoma in situ, **e** positive lymph node, **f** positive surgical margin

For pathological T stage, patients with SV-UCB had a significant lower rate of ≤ pT2 disease (44.7% vs. 72.0%) (OR, 0.41; 95% CI, 0.23–0.71; p = 0.001) (Fig. 2a) and higher rate of ≥ pT3 disease (35.0% vs. 18.5%) (OR, 2.06; 95% CI, 1.64–2.59; p < 0.001) (Fig. 2b). However, patients with SV-UCB had a lower rate of concomitant carcinoma in situ (12.1% vs. 33.0%) (OR, 0.25; 95% CI, 0.09–0.72; p = 0.010) (Fig. 2d). In terms of other variables, there was no significant difference for high grade (92.3% vs. 71.9%) (OR, 2.61; 95% CI, 0.02–286.71; p = 0.690) (Fig. 2c), positive lymph node (22.9% vs. 28.1%) (OR, 0.79; 95% CI, 0.40–1.59; p = 0.520) (Fig. 2e), positive surgical margin (4.2% vs. 8.9%) (OR, 0.55; 95% CI, 0.15–1.98; p = 0.360) (Fig. 2f) between patients with SV-UCB and C-UCB.

### Survival outcomes

**Fig. 3:**
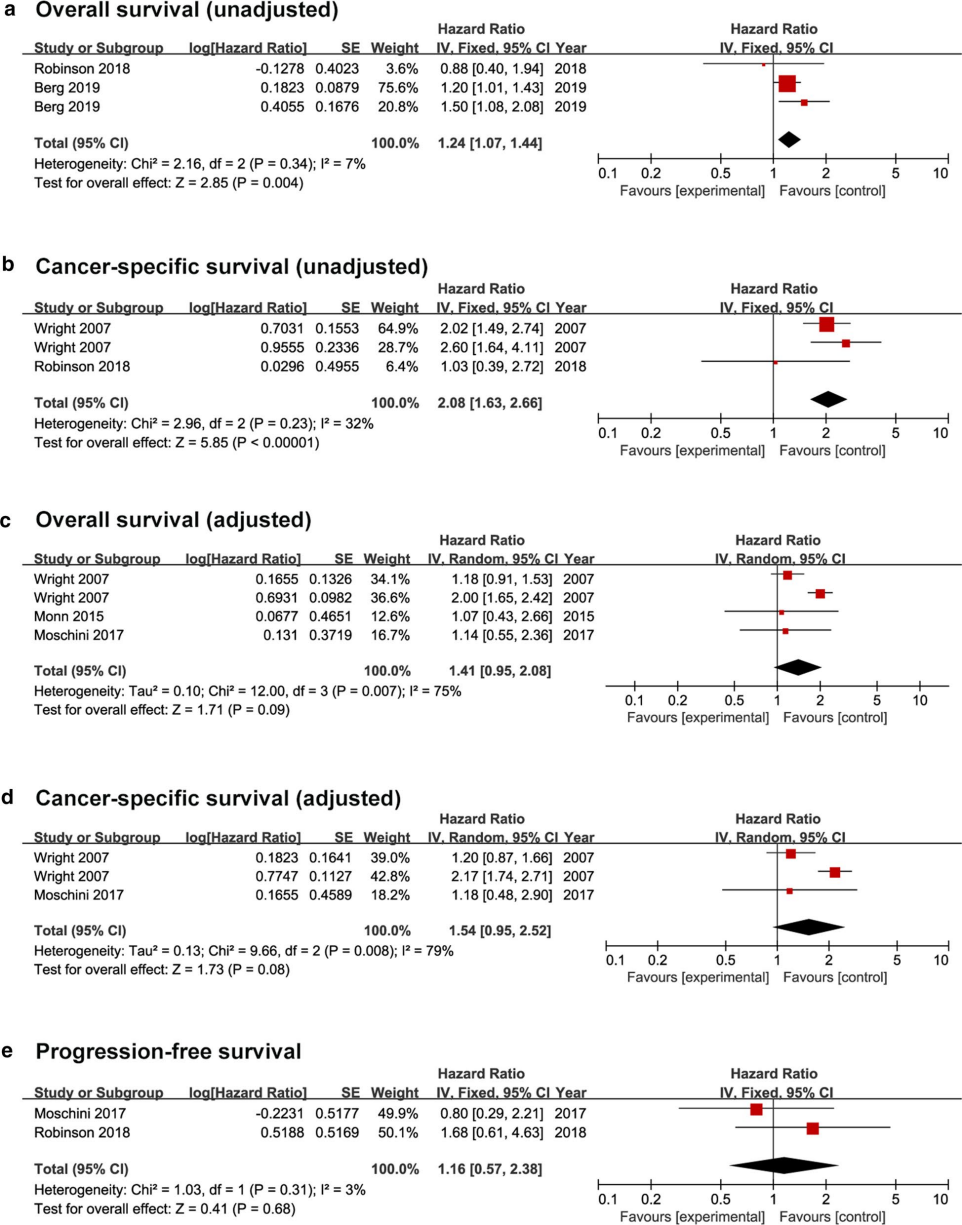
Forest plots of the survival outcomes. **a** overall survival (unadjusted), **b** cancer-specific survival (unadjusted), **c** overall survival (adjusted), **d** cancer-specific survival (adjusted), **e** progression-free survival

For OS and CSS, the meta-analyses were separately preformed with unadjusted and adjusted data. Appling the unadjusted statistic values, patients with SV-UCB had a significant inferior OS (HR, 1.24; 95% CI, 1.07–1.44; p = 0.004) (Fig. 3a) and CSS (HR, 2.08; 95% CI, 1.63–2.66; p < 0.001) (Fig. 3b). However, with the adjusted statistic values, the OS (HR, 1.41; 95% CI, 0.95–2.08; p = 0.090) (Fig. 3c) and CSS (HR, 1.54; 95% CI, 0.95–2.52; p = 0.080) (Fig. 3d) were similar for patients with SV-UCB and C-UCB. For PFS, one study has reported unadjusted result [10] and one study has reported adjusted result [11]. After merging these results, we found that sarcomatoid variant had no influence on PFS for UCB (HR, 1.16; 95% CI, 0.57–2.38; p = 0.680) (Fig. 3E).

### Neoadjuvant chemotherapy and adjuvant chemotherapy

**Fig. 4:**
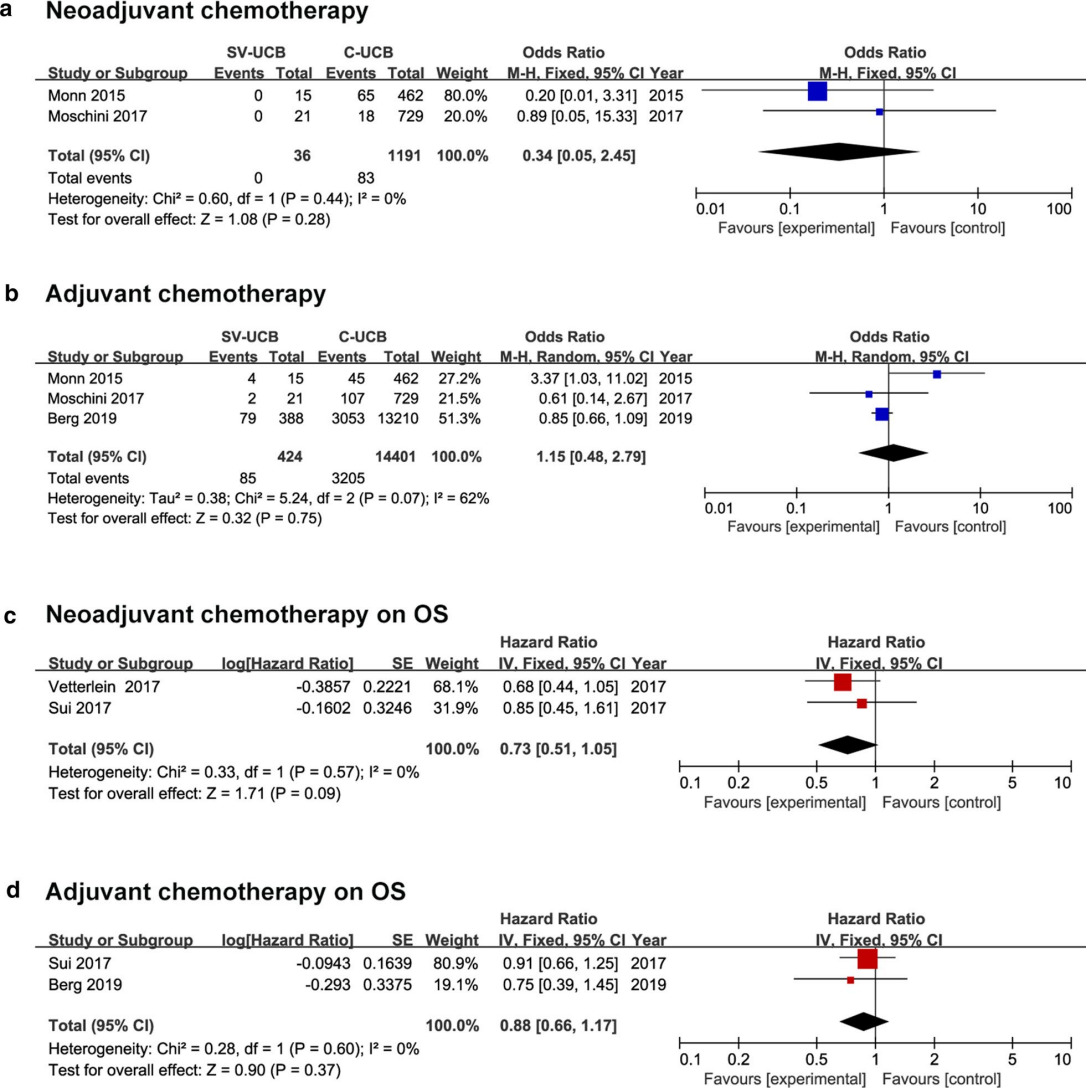
Forest plots of neoadjuvant chemotherapy and adjuvant chemotherapy. **a** Rate of neoadjuvant chemotherapy, **b** rate of adjuvant chemotherapy, **c** neoadjuvant chemotherapy on OS, **d** adjuvant chemotherapy on OS

Compared with those with C-UCB, patients with SV-UCB had a lower rate of NAC (0.0% vs. 7.0%) and AC (20.0% vs. 22.3%) administration, however, the differences got no statistical significance (NAC: OR, 0.34; 95% CI, 0.05–2.45; p = 0.280 and AC: OR, 1.15; 95% CI, 0.48–2.79; p = 0.750) (Fig. 4a, b). For patients with SV-UCB, NAC (HR, 0.73; 95% CI, 0.51–1.05; p = 0.090) and AC (HR, 0.88; 95% CI, 0.66–1.17; p = 0.370) seemed to have no benefit on OS, the merged results were based on adjusted data (Fig. 4c, d).

### Quality assessment and qualitative risk of bias

The results of quality evaluation for included studies were presented in Additional file 2: Table S1. Of them, one study obtained 6 stars, two studies obtained 7 stars, five studies obtained 8 stars. The evaluation of the quality of evidence of each comparison with the GRADE system was presented in Table 3. There were 15 comparison. Certainty was moderate in pathologic T stage 3 and higher, concomitant carcinoma in situ, unadjusted CSS, and was low in pathologic T stage 2 and lower, unadjusted OS. It was very low for other comparisons.

**Table 3:** Grading of Recommendations, Assessments, Developments, and Evaluation (GRADE) quality assessment of evidence for each comparison

Number of studies	Study design	Certainty assessment					Number of patients		Effect		Certainty	Importance
		Risk of bias	Inconsistency	Indirectness	Imprecision	Other considerations	SV-UCB	C-UCB	Relative (95% CI)	Absolute (95% CI)		
Pathologic T stage 2 and lower												
4	Observational studies	Not serious	Serious	Not serious	Not serious	Strong association	156/349 (44.7%)	34,499/47,936 (72.0%)	OR 0.41 (0.23–0.71)	207 fewer per 1000 (from 348 to 74 fewer)	⨁⨁◯◯ LOW	CRITICAL
Pathologic T stage 3 and higher												
4	Observational studies	Not serious	Not serious	Not serious	Not serious	Strong association	122/349 (35.0%)	8862/47,936 (18.5%)	OR 2.06 (1.64–2.59)	134 more per 1000 (from 86 to 185 more)	⨁⨁⨁◯ MODERATE	CRITICAL
High grade												
2	Observational studies	Not serious	Serious	Not serious	Serious	None	24/26 (92.3%)	395/549 (71.9%)	OR 2.61 (0.02–286.71)	151 more per 1000 (from 671 fewer to 279 more)	⨁◯◯◯ VERY LOW	CRITICAL
Concomitant carcinoma in situ												
2	Observational studies	Not serious	Not serious	Not serious	Not serious	Strong association	4/33 (12.1%)	316/959 (33.0%)	OR 0.25 (0.09–0.72)	220 fewer per 1000 (from 287 to 68 fewer)	⨁⨁⨁◯ MODERATE	CRITICAL
Positive lymph node												
3	Observational studies	Not serious	Not serious	Not serious	Serious	None	11/48 (22.9%)	400/1421 (28.1%)	OR 0.79 (0.40–1.59)	45 fewer per 1000 (from 146 fewer to 102 more)	⨁◯◯◯ VERY LOW	CRITICAL
Positive surgical margin												
3	Observational studies	Not serious	Not serious	Not serious	Serious	None	2/48 (4.2%)	126/1421 (8.9%)	OR 0.55 (0.15–1.98)	38 fewer per 1000 (from 74 fewer to 73 more)	⨁◯◯◯ VERY LOW	CRITICAL
Overall survival (unadjusted)												
2	Observational studies	Not serious	Not serious	Not serious	Not serious	None	–	–	HR 1.24 (1.07–1.44)	1 fewer per 1000 (from 1 to 1 fewer)	⨁⨁◯◯ LOW	IMPORTANT
Cancer-specific survival (unadjusted)												
2	Observational studies	Not serious	Not serious	Not serious	Not serious	Strong association	–	–	HR 2.08 (1.63–2.66)	2 fewer per 1000 (from 3 to 2 fewer)	⨁⨁⨁◯ MODERATE	IMPORTANT
Overall survival (adjusted)												
3	Observational studies	Not serious	Serious	Not serious	Serious	None	–	–	HR 1.41 (0.95–2.08)	1 fewer per 1000 (from 2 to 1 fewer)	⨁◯◯◯ VERY LOW	CRITICAL
Cancer–specific survival (adjusted)												
2	Observational studies	Not serious	Serious	Not serious	Serious	None	–	–	HR 1.54 (0.95–2.52)	2 fewer per 1000 (from 3 to 1 fewer)	⨁◯◯◯ VERY LOW	CRITICAL
Progression-free survival												
2	Observational studies	Not serious	Not serious	Not serious	Serious	None	–	–	HR 1.16 (0.57–2.38)	1 fewer per 1000 (from 2 to 1 fewer)	⨁◯◯◯ VERY LOW	IMPORTANT
Neoadjuvant chemotherapy												
2	Observational studies	Not serious	Not serious	Not serious	Serious	None	0/36 (0.0%)	83/1191 (7.0%)	OR 0.34 (0.05–2.45)	45 fewer per 1000 (from 66 fewer to 85 more)	⨁◯◯◯ VERY LOW	CRITICAL
Adjuvant chemotherapy												
3	Observational studies	Not serious	Serious	Not serious	Serious	None	85/424 (20.0%)	3205/14,401 (22.3%)	OR 1.15 (0.48–2.79)	25 more per 1000 (from 102 fewer to 221 more)	⨁◯◯◯ VERY LOW	CRITICAL
Neoadjuvant chemotherapy on OS												
2	Observational studies	Not serious	Not serious	Not serious	Serious	None	–	–	HR 0.73 (0.51–1.05)	1 fewer per 1000 (from 1 to 1 fewer)	⨁◯◯◯ VERY LOW	CRITICAL
Adjuvant chemotherapy on OS												
2	Observational studies	Not serious	Not serious	Not serious	Serious	None	–	–	HR 0.88 (0.66–1.17)	1 fewer per 1000 (from 1 to 1 fewer)	⨁◯◯◯ VERY LOW	CRITICAL

*SV-UVB* sarcomatoid variant urothelial carcinoma of the bladder, *C-UCB* conventional urothelial carcinoma of the bladder, *CI* confidence interval, *OR* odd ratio, *HR* hazard ratio, *OS* overall survival

## Discussion

More recently, systematic reviews have described the prognostic significance of histological variants in UCB, and the diagnostic, therapeutic management of UCB with histological variants [1, 19]. Indeed, these systematic reviews provided much important information for urologists and oncologists. However, in order to perform a more comprehensive overview, they analyzed all types of histological variants together. Inevitably, the limited evidence for specific histological variant was presented in these studies, and meta-analysis was not performed.

Sarcomatoid variant is a rare histologic variant of UC, comprising less than 1% of all UCB. Though former evidences supported that SV-UCB was aggressive, prone to present at an advanced stage and was associated with poor long-term survival [3, 8, 9], many studies have denied the prognosis significance of sarcomatoid variant in UCB [10–12]. The behavior of SV-UCB and its treatment guidelines are not well characterized. In the present study, we systematically reviewed the relevant literatures and performed meta-analyses with available data comparing the clinicopathological features and survival outcomes between SV-UCB and C-UCB.

According to our findings, compared to C-UCB, patients with SV-UCB trend to experience a higher pathological T stage, which may be associated with a poor survival outcome. However, concomitant carcinoma in situ was more often identified in patients with urothelial cell carcinoma, which was inconsistent with the result of pathological T stage. In terms of other variables, there was no significant difference in tumor grade, positive lymph node, and positive surgical margin. When analyzing pathological T stage, 349 patients with SV-UCB were included, however, for other comparisons, only 26–48 patients with SV-UCB were included. Moreover, concomitant carcinoma in situ, positive lymph node, positive surgical margin were low-frequency events. Combining these considerations, the result of pathological T stage was more reliable, so we prone to believe that SV-UCB is associated with more advanced disease.

A high pathological T stage in patients with SV-UC may transform to a worse prognosis. According to our meta-analyses, compared with those with C-UCB, patients with SV-UCB had a significant inferior OS and CSS using unadjusted statistical data. However, after adjusted with other clinicopathological features (i.e. age, sex, pathological T stage, positive lymph node or surgical margin, NAC, AC), sarcomatoid variant failed to be independent prognosis predictor for patients with UCB. According to previous methodology [20], when considering that the p value is approaching the significance borderline and the CI range is wide, there is still a high probability that an independent prognostic factor exists. It is thought to be related to the lack of sample enrollment; if a sufficient number of samples are achieved, sufficient statistical power might be ensured. Hence, the present study raised the possibility that sarcomatoid variant might be an independent prognosis predictor in patients with UCB.

Due to the rarity of SV-UCB, the relevant randomized controlled trials were hardly performed. Therefore, there is no standard treatment for this disease. For patients with sarcomatoid variant, the treatment mainly extrapolated from the strategy for C-UCB [5]. Actually, there is controversy about whether surgery alone or multimodality would be most effective. Several observational studies have provided insights into the treatment of SV-UCB. Wang et al. [18] have reported that aggressive multi-modal treatment in 3 out of 14 patients achieved a complete response and long-term survival. Of the 3 patients, 1 received neoadjuvant chemotherapy and 2 received 4 cycles of cisplatin and gemcitabine adjuvant chemotherapy. Robinson et al. [10] have described multimodal treatment in 4 out of 12 patients with surgically treated SV-UCB. Two patients who received the adjuvant gemcitabine and cisplatin were still alive at 118 and 8 months, respectively. One patient received neoadjuvant radiotherapy, but died 45 months later. Another received neoadjuvant chemotherapy and died 9 months after surgery. Compared with single-center series, studies based on database cohorts reported different outcomes, which may due to the differences in sample size and practice patterns (cystectomy, radiation, and chemotherapy). Considering the rarity of this tumor, a multidisciplinary approach is highly recommended at referral centers.

Meanwhile, since limited cases were reported, the evidence of systemic chemotherapy for patients with SV-UCB is insufficient. In a conference abstract, Black et al. [21] have described case series with SV-UCB, 11 of them had NAC, and 34 of them were managed by surgery alone. Though no survival benefit of NAC was identified, the rate of downstaging to pT0 after NAC was 45% at the time of radical cystectomy. Spiess et al. [22] have reported 7 (41%) of 17 cases with SV-UCB were treated by NAC. Several chemotherapeutic regimens were applied, and cancer specific mortality was 65% with an average follow-up of 21 months. A few cases have been reported of complete remission after neoadjuvant chemoradiotherapy [23, 24]. In the present study, we only included comparative studies. The endpoints included rate of NAC and AC administration between SV-UCB and C-UCB, survival benefit of NAC and AC for SV-UCB. Based on our results, compared with those with C-UCB, patients with SV-UCB had a lower rate of NAC (0.0% vs. 7.0%) and AC (20.0% vs. 22.3%) administration, however, the differences got no statistical significance. Although several studies have identified that SV-UCB can achieved a longer survival with NAC or AC compared to surgery alone, the difference was not significant. Based on the adjusted data, NAC or AC was not independently associated with OS in SV-UCB. Multiple epithelial-mesenchymal transition (EMT) pathways have been studied in SV-UCB [25, 26]. An EMT-targeted program could be an effective therapeutic strategy for these malignances. Since high EMT scores was correlated with distinct immunophenotypes and increased expression of immunosuppressive molecules in lung cancer, underlying mechanisms of EMT-related immunosuppression could be utilized. New investigations found that tumor with sarcomatoid variant may express higher percentage PD-1/PD-L1 than those without sarcomatoid variant, suggesting that tumor with sarcomatoid variant may be more suitable for anti-PD-1/PD-L1 therapy [27]. However, further researches were needed to verify these speculations.

As far as we know, the current study is the first meta-analysis comparing the clinicopathological features and survival outcomes between SV-UCB and C-UCB. However, several inevitable limitations existed. Due to the rarity of sarcomatoid variant in UCB, the related studies were relatively insufficient and sample size was small, which may affect the data quality. All included studies were observational studies with retrospective and non-randomized design, the potential selection bias and uncontrolled confounding factors may affect the results. Moreover, the definition of SV-UCB was non-uniform among included studies. The GRADE approach was applied to assess the certainty of evidence, it was moderate for pT stage ≥ 3, concomitant carcinoma in situ, but the other comparisons were low or very low. Despite these limitations, the present study may provide important information for clinicians in the process of managing SV-UCB and decision-making. In order to provided robust recommendation with high-quality evidence, well-designed multi-institutional studies are needed, particularly in determining independent prognostic role and specifying optimal treatment. Additionally, a uniform definition of sarcomatoid variant in UCB is warranted, and detailed description of sarcomatoid variant in pathological report is recommended for pathologists.

## Conclusions

Our findings indicated that SV-UCB was associated with more advanced disease, especially for higher pathological T stage. Compared with C-UCB, sarcomatoid variant in UCB was associated with inferior survival outcomes. It was very likely that sarcomatoid variant might be an independent prognosis predictor in patients with UCB. NAC and AC had no survival benefit for SV-UCB. Our results may help clinicians in the process of managing SV-UCB and decision-making. Nevertheless, duo to the lacking evidence, the optimal management for SV-UCB is not settled. In order to provided robust recommendation with high-quality evidence, well-designed multi-institutional studies are needed.

## Supplementary information

**Additional file 1.**

**Additional file 2: Table S1.** Newcastle–Ottawa Scale for risk of bias assessment of studies included in the meta-analysis.

## Data Availability

All the data (pooled odds ratios or hazard ratios with 95% confidence intervals) used to support the findings of this study are included within the article. Please contact author for data requests.
