# Supplementary material for: Sarcomatoid variant urothelial carcinoma of the bladder: a systematic review and meta-analysis of the clinicopathological features and survival outcomes
Source: Cancer Cell Int. 2020 Nov 14;20:550. doi: 10.1186/s12935-020-01626-9 (PMC7666462; doi:10.1186/s12935-020-01626-9)
Supplement: Supplementary file 2 — Additional file 2: Table S1. Newcastle–Ottawa Scale for risk of bias assessment of studies included in the meta-analysis. [file 12935_2020_1626_MOESM2_ESM.docx]

**Table S1.** Newcastle-Ottawa Scale for risk of bias assessment of studies included in the meta-analysis.

| Studies | Selection | | | | Comparability | Outcome | | | Overall |
| --- | --- | --- | --- | --- | --- | --- | --- | --- | --- |
|  | Representativeness of exposed cohort | Selection of nonexposed | Ascertainment of exposure | Outcome not present at start |  | Assessment of outcome | Adequate follow-up length | Adequacy of follow-up |  |
| Berg (2019) | ★ | ★ | ★ | ★ | ★ | ★ | ★ | ★ | 8 |
| Robinson (2018) | ★ | ★ | ★ | ★ | ★ | ★ | ★ | ☆ | 7 |
| Vetterlein (2017) | ★ | ★ | ★ | ★ | ★ | ★ | ★ | ★ | 8 |
| Sui (2017) | ★ | ★ | ★ | ★ | ★ | ★ | ★ | ★ | 8 |
| Moschini (2017) | ★ | ★ | ★ | ★ | ★ | ★ | ★ | ★ | 8 |
| Monn (2015) | ★ | ★ | ★ | ★ | ★ | ★ | ★ | ☆ | 7 |
| Wang (2011) | ★ | ★ | ★ | ★ | ★ | ★ | ☆ | ☆ | 6 |
| Wright (2007) | ★ | ★ | ★ | ★ | ★ | ★ | ★ | ★ | 8 |
